# Precipitation of NH_4_UO_2_PO_4_·3H_2_O—Solubility and Structural Comparison with Alkali Uranyl(2 +) Phosphates

**DOI:** 10.6028/jres.093.148

**Published:** 1988-08-01

**Authors:** Milenko Marković, Nevenka Pavković, Neven D. Pavković

**Affiliations:** “Rudjer Bošković” Institute, Zagreb, Yugoslavia; ADAHF, National Bureau of Standards Gaithersburg, MD 20899; Faculty of Science, Zagreb, Yugoslavia; “Rade Končar” Selfmanagement Corporation, Zagreb, Yugoslavia

**Keywords:** alkali uranyl(2+)phosphates, ammonium uranyl(2+)phosphate, precipitation, solubility product, unit-cell dimensions, x-ray diffraction pattern

## Abstract

Precipitates formed in the system UO_2_(NO_3_)_2_-NH_4_OH-H_3_PO_4_-H_2_O, aged for 30 days at 298 K, were studied. The precipitates were characterized by chemical and thermogravimetric analyses, x-ray powder diffraction, infrared spectroscopy, polarized light microscopy, and by their fluorescent properties. The precipitation boundary was established tindallometrically and microscopically. On the basis of these measurements, the stability conditions, structural parameters, and solubility of the tetragonal polymorph of NH_4_[UO_2_PO_4_]·3H_2_O were determined. This compound shows a close structural relationship with H_3_O[UO_2_PO_4_]·3H_2_O (space group *P*_4_/*ncc*) and alkali uranyl(2+)phosphates polyhydrates M[UO_2_PO_4_]·*n*H_2_O (*n* =4 for M=Li; *n* =3 for M=Na, K, Rb and *n* =2.5 for M=Cs). The unit-cell dimensions determined for NH_4_UO_2_PO_4_·3H_2_O are: *a*=*b*=7.02 Å, *c*=18.08 Å (*P*_4_/*ncc*). The thermodynamic solubility product constant, *K*_s_*=a*(NH_4_^+^)×*a*(UO_2_^2+^)×*a*(PO_4_^3−^), for NH_4_UO_2_PO_4_·3H_2_O was determined: log *K*_s_= −26.50±0.09. The *K*_s_ values of M[UO_2_PO_4_]·*n* H_2_O (at ionic strength, *I*=0.23 mol dm^−3^) calculated from previously published experimental data by using correct stability constants of uranyl(2+)phosphate complexes are:

log *K*_s_=−22.61±0.08 for M=Na;
log *K*_s_= −23.92±0.12 for M=K;
log *K*_s_= −24.13±0.19 for M=Rb;
log *K*_s_= −23.80±0.20 for M=Cs; and
log *K*_s_= −24.74±0.10 for M=NH_4_,

showing that NH_4_UO_2_PO_4_·3H_2_O is less soluble than corresponding alkali uranyl(2+)phosphates.

## Introduction

The formation of uranyl(2 +)phosphates (UO_2_HPO_4_·4H_2_O and (UO_2_)_3_(PO_4_)_2_·8H_2_O) and alkali uranyl(2+)phosphates (MUO_2_PO_4_·*n*H_2_O; M=Li, Na, K, Rb, Cs; 4 ⩾*n* ⩾2.5) by spontaneous precipitation from supersaturated solutions and the stability of uranyl phosphate complexes has been described in some of our previous papers [1–5]. These compounds are important for the production of uranium from low-grade phosphate ores and in fuel reprocessing [6–8].

Precipitation conditions of ammonium uranyl(2 +)phosphate can be of interest for the separation of uranium as a secondary product in the production of monoammonium phosphate (additive of fertilizers) [9]. Three polymorphs of NH_4_UO_2_PO_4_·3H_2_O(s) are known [10,11]. The solubility product of one of these compounds has been determined at undefined ionic strength [12,13] and at an ionic strength of 0.23 mol dm^−3^ [14] using inaccurate association and stability constants for phosphoric acid and uranyl phosphate complexes, respectively.

This paper describes the formation of different precipitates in the system UO_2_(NO_3_)_2_-NH_4_OH-H_3_PO_4_-H_2_O at 298 K. These precipitates were characterized by chemical and physical methods. The stability region for the precipitated tetragonal polymorph of NH_4_UO_2_PO_4_·3H_2_O was established as a function of reactant concentrations, and its solubility product constant was determined. The structure of this polymorph was compared to that of hydrogen uranyl(2 +)phosphate [2,15] and alkali uranyl(2+)phosphates [3,4]. The solubility data for NH_4_UO_2_PO_4_·3H_2_O and MUO_2_PO_4_·*n*H_2_O obtained by Vesely, Pekarek, and Abbrent [14] were recalculated in this paper using a proper set of constants to obtain solubility products, and they were compared with our data.

## Experimental Section

Stock solutions were prepared by dissolving the following P.A. chemicals in triply distilled water: UO_2_(NO_3_)_2_, H_3_PO_4_, and NH_4_OH (Merck,^2^ Darmstadt). Standardization of solutions was performed by using classical analytical methods [16,17].

Precipitation in the system UO_2_(NO_3_)_2_-NH_4_OH-H_3_PO_4_-H_2_O (at 298 K) was performed at constant uranyl(2+)nitrate concentration, 1 × 10^−3^ mol dm^−3^; the concentrations of NH_4_OH varied from 5 × 10^−5^ to 3.2 mol dm^−3^ and phosphoric acid from 5 × 10^−3^ to 1 mol dm^−3^. The samples were prepared by mixing UO_2_(NO_3_)_2_ solution with an equal volume of NH_4_OH + H_3_PO_4_ solution. Approximately 400 samples were prepared to define precipitation and phase boundaries. One day and 30 days after mixing the reactant solutions, the samples were examined in detail. The pH was measured with the Radiometer equipment: electrode GK 2302 C and pH-meter Mo 26. The precipitation boundary (the line that separates the region of precipitation from the region of clear solutions) was determined tyn-dallometrically and microscopically. The morphology of the precipitates was examined in white, polarized and UV light under an Orthoplan microscope (Leitz, Wetzlar). Selected precipitates were characterized by means of chemical and thermogravimetric analyses (TGA), x-ray powder diffraction patterns (XRD) and infrared (IR) spectra. The phase boundaries (lines that separate the regions in which different solid phases precipitate) were determined on the basis of these data.

The solid phase was chemically analyzed for uranium, phosphorus and nitrogen. Uranium was precipitated with (NH_4_)_2_HPO_4_, heated at 1373 K and weighed as U_2_O_3_P_2_O_7_ [18]. Phosphorus was determined gravimetrically by precipitation with ammonium molybdate [19] and spectrophotometrically as phosphovanadomolybdato complex [19]. Nitrogen was determined by chemical microanalysis. The water content was determined thermogravimetrically (Cahn RG recording electromicrobalance).

X-ray diffraction patterns were recorded on a Phillips x-ray diffractometer with a proportional counter, using graphite monochromated CuKα radiation. The x-ray patterns were calibrated with graphite as the internal standard [10] with a unit-cell *a* =2.463 Å, *c* =6.714 Å (λ= 1.54178 Å). Relative intensities, *I*_rel_, are given as peak heights. IR spectra (600 to 3600 cm^−1^) were obtained using a Perkin-Elmer Mo-221 spectrophotometer and the standard KBr pellet technique.

## Results

**Figure 1:**
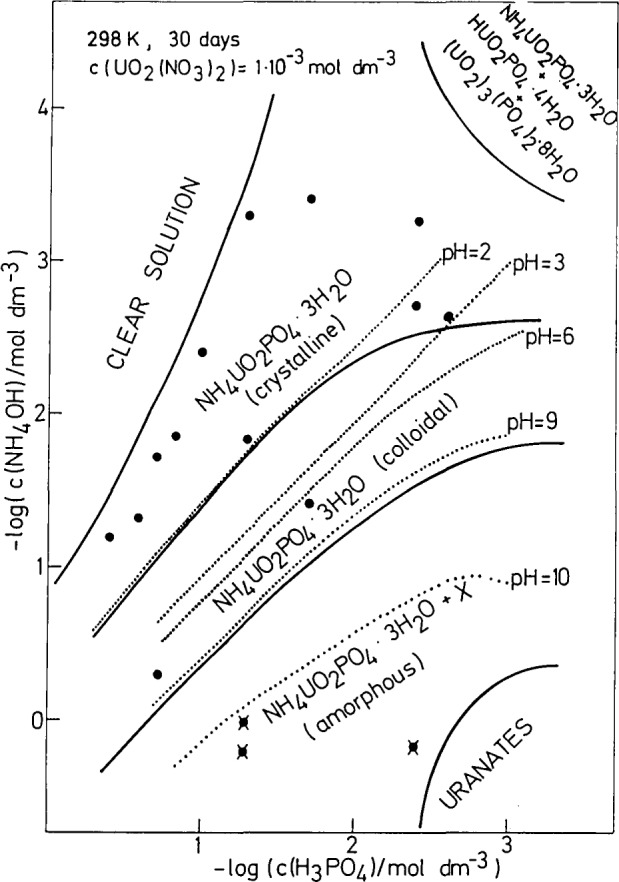
Precipitation diagram for the system UO_2_(NO_3_)_2_-NH_4_OH-H_3_PO_4_-H_2_O aged for 30 days. Precipitation and phase boundaries are denoted by full lines and iso-pH curves by dotted lines. In the samples (●,✸), the solid phase was identified by XRD, IR, chemical, and TG analysis and found to be NH_4_UO_2_PO_4_·3H_2_O (●) and an undefined compound (X).

The concentration diagram of UO_2_(NO_3_)_2_-NH_4_OH-H_3_PO_4_-H_2_O systems aged for 30 days is presented in figure 1. The precipitation and phase boundaries (full lines) and iso-pH curves (dotted lines) are shown. Only the experimental points representing samples in which solid phase was fully examined (XRD, IR, TGA, chemical analysis) are shown in figure 1 (filled circles). Chemical and TG analyses revealed that the solid phase was NH_4_UO_2_PO_4_·3H_2_O:
%U%P%N% H_2_OFound:54.28–54.607.06–7.123.16–3.2512.30–12.40Theoretical:54.467.093.2012.36TGA showed the loss of 2.4±0.2 mol H_2_O up to 353 K and an additional 0.6±0.2 mol loss in the interval from 353 to 403 K. Transformation of anhydrous NH_4_UO_2_PO_4_ to UO_2_HPO_4_ (loss of NH_3_) starts at 450 K. The IR spectrum of NH_4_UO_2_PO_4_·3H_2_O showed characteristic phosphate and uranyl vibrations [20].

In table 1 are given observed *d*-values (*d*_obsd_) and the relative intensities (*I*_rel_) for NH_4_UO_2_PO_4_·3H_2_O obtained by XRD. Comparison of the x-ray powder pattern of NH_4_UO_2_PO_4_·3H_2_O with those of the H_3_O[UO_2_PO_4_]·3H_2_O [2,15] confirms a close structural relationship among them (*P*_4_/*ncc* space group, structure: metatorbernite); the (*h* 00) and (00*l*) reflections were used to calculate the unit cell parameters for NH_4_UO_2_PO_4_·3H_2_O: *a =b* =7.02(1) Å, and *c* = 18.08(4) Å. The *hkl* indices and *d*_calcd_ values (table 1) are calculated on the basis of unit-cell parameters by using computer programs [21,22]. The excellent agreement between observed and calculated *d* values (table 1) indicate a pure tetragonal polymorph NH_4_UO_2_PO_4_·3H_2_O.

NH_4_UO_2_PO_4_·3H_2_O crystallizes in the broad concentration range pH⩽2 (fig. 1). Its crystals were in the form of squarish platelets showing an intense green fluorescence. In the region where a small increase in the ratio *c*(NH_4_OH)/*c*(H_3_PO_4_) (at constant *c*(H_3_PO_4_)) causes a steep jump in the pH values of successive samples (from 2 to 9), stable colloidal particles obtained. At low concentrations of NH_4_OH and H_3_PO_4_ mixtures of NH_4_UO_2_PO_4_·3H_2_O with H_3_O[UO_2_PO_4_]·3H_2_O and (UO_2_)_3_(PO_4_)_2_·8H_2_O were found. At pH>9 mixtures of amorphous NH_4_UO_2_PO_4_·3H_2_O (prevailing solid phase) and an undefined compound (designated by X in fig. 1) precipitated. The chemical identification of X was not possible due to its extremely small presence (less than 5 %).

In table 2 are given the concentrations of all components in the solutions equilibrated with NH_4_UO_2_PO_4_·3H_2_O(s) (points along the precipitation boundary). The ionic concentration product, *K*_s_*=c*(NH_4_^+^)*×c*(UO_2_^2+^)×*c*(PO_4_^3−^), expressed in greater detail form is

$$K_{s}=\frac{c{\left({\text{UO}}_{2}\right)}_{\text{soln}}\times c{\left({\text{NH}}_{4}\right)}_{\text{soln}}\times c(H_{3}{\text{PO}}_{4})}{K_{13}\times K_{12}\times K_{1}\times a^{3}(H^{+})}\times \sum_{i=0}{\sum_{j=0}\left(\frac{\beta_{ij}\times c{\left(H_{3}{\text{PO}}_{4}\right)}^{i+j}}{a{\left(H^{+}\right)}^{j}}\right)}^{-1}.$$

**Figure 2:**
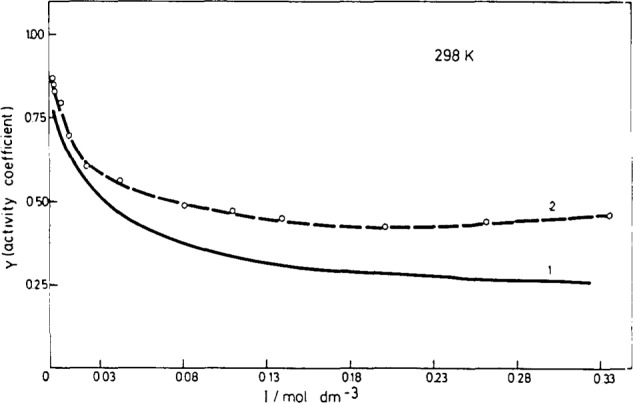
Calculated activity coefficients [26] for the ions with ±2 valence charge (curve 1) and experimentally determined activity coefficients for UO_2_^2+^[27] (curve 2) as a function of the ionic strength.

In this equation *c*(UO_2_)_soln_ and *c*(NH_4_)_soln_ are the total concentrations of uranyl and ammonium species in the solution, respectively. *K*_13_, *K*_12_, and *K*_1_ are the association constants of phosphoric acid [23–25] (table 3, equilibria 1–3) and *β_ij_* are the stability constants of different uranyl phosphate complexes [5] (table 3, equilibria 4–7). The calculations were performed using a computer program designed on the basis of the procedure published earlier (ref. [5], eqs 1–5). The input data for the program were the concentrations of all components in the solution (table 2) and the values of thermodynamic equilibrium constants at 298 K (table 3, equilibria 1–7). The ionic strength, *I*, defined as *I* =0.5 Σ*cz*^2^ (*c* and *z* are the concentration and valence charge of the ion, respectively) was calculated by an iterative procedure (iterations until the change was less than ± 1 %). Consequently, the values of the equilibrium constants at *I* = 0 were calculated from thermodynamic equilibrium constants by using the values of the activity coefficients (*y*) of the ions at corresponding ionic strengths. Activity coefficients (at 298 K) of all ions (except UO_2_^2+^) were calculated by using the Davies equation [26]: $\log\;y=-0.509z^{2}[\sqrt{I}/(\sqrt{I}+\;1)-0.2I]$. For uranyl(2 +) ions the activity coefficients determined by Brusilovsky [27] were used. In figure 2 is presented the dependence of the activity coefficients on the ionic strength: for the ions with valence charge 2 the curve was calculated by using the Davies equation (curve 1) and for the uranyl(2 +) ions it was constructed by using the experimental values [27] (curve 2). The difference between these two curves is considerable.

The calculated values of the solubility products (log *K*_s_) at the corresponding ionic strengths and the thermodynamic values [log *K*_s_ (*I* =0)] are presented in table 2; log *K*_s_ (*I* = 0) has at 298 K an average value of −26.50±0.09 (table 3, equilibrium 9).

## Discussion

Ammonium uranyl(2 +)phosphate trihydrate precipitates as the only solid phase in a broad concentration range of the reactants (fig. 1). On the contrary, in the presence of alkali ions, mixtures consisting of hydrogen and alkali uranyl(2+)phosphates prevail [3,4]. These results can be explained by the greater sorption affinity of NH_4_^+^ on uranylhydrogen(2 +)phosphate tetrahydrate as compared to that of alkali cations [28].

In order to compare the solubilities of ammonium and alkali uranyl(2 +)phosphates we recalculated the solubility data of Vesely, Pekarek, and Abbrent [14] (experiments performed at *I*=0.23 mol dm^−3^) by using reported association constants of phosphoric acid [23–25] (table 3, equilibria 1–3) and stability constants of uranyl(2 +)phosphate complexes [5] (table 3, equilibria 4–7) corrected from *I* =0 to *I* =0.23 mol dm^−3^ (by using experimental activity coefficients of UO_2_^2+^ ions [27]). The average values of ionic activity and concentration products are listed (table 3, equilibria 9–13). The solubilities of different uranyl phosphate compounds depend on the cationic species in the structure. The ionic product constants (*K*_s_) increase as follows: *K*_s_(NH_4_) < *K*_s_(Rb) < *K*_s_(K) < *K*_s_(Cs) < *K*_s_(Na).

Our experimentally determined *K*_s_(*I* =0) values of NH_4_UO_2_PO_4_·3H_2_O and KUO_2_PO_4_·3H_2_O [3] (table 3, equilibria 9 and 11) corrected to *I* =0.23 mol dm^−3^ shows an excellent agreement with the corresponding values recalculated from the data [14] originally determined at *I* =0.23 mol dm^−3^. This confirms the accuracy of the stability constants of the uranyl phosphate complex species [5] and the experimental precision of the solubility data [3,14]. The *K*_s_ values determined and those recalculated in this work are in disagreement with the values given by Klygin et al. [13] and Muraveva et al. [29]. These authors did not consider the uranyl(2 +)phosphate complex formation. The value of *K*_s_ for KUO_2_PO_4_·3H_2_O determined by Chukhlantsev and Stepanov [12] is a hundred times higher than ours, but their solubility product constant of NH_4_UO_2_PO_4_·3H_2_O (at undefined ionic strength) is similar, (log *K*_s_= −26.36) [12], to the value determined in this work (at *I* =0). It seems that (a) experimental uncertainties and (b) calculations which do not take into account complex species compensate each other, giving a value of *K*_s_ for NH_4_UO_2_PO_4_·3H_2_O similar to the one we determined. Recalculation of their data [12] is not possible because the analyses of the equilibrated solutions were incomplete.

**Figure 3:**
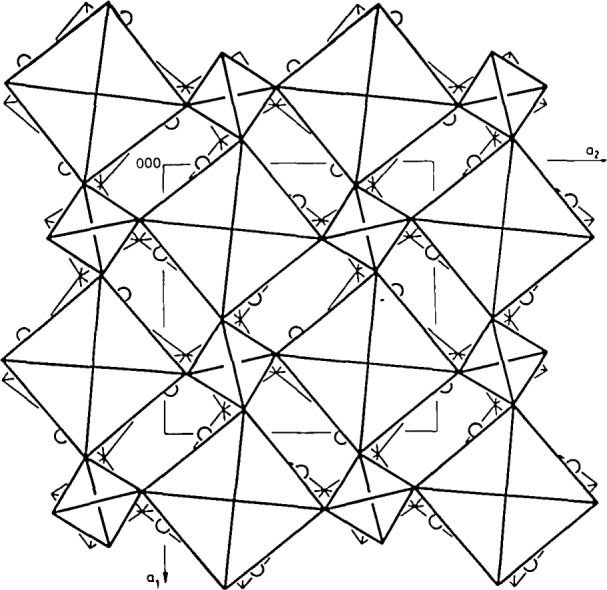
The infinite layers of [UO_2_PO_4_]^−^ (composed of octahedra and tetrahedra) and water layers in the structure of H_3_O[UO_2_PO_4_]·3H_2_O [15]. The water molecules in the water layer are designated by the open circles.

The x-ray powder pattern of NH_4_[UO_2_PO_4_]·3H_2_O reveals a close structural relationship with the series M[UO_2_PO_4_]·*n*H_2_O (*n* =4 for M=Li; *n* =3 for M=H_3_O, Na, K, Rb; and *n* =2.5 for M=Cs). The crystal structure of M[UO_2_PO_4_]·*n*H_2_O (fig. 3, structure of H_3_O[UO_2_PO_4_]·3H_2_O [15]) reveals packing arrangements of infinite layers of octahedra and tetrahedra and water layers containing M (H_3_O^+^, alkali or NH_4_^+^) ions. Uranium exhibits an octahedral coordination. The PO_4_ tetrahedron acts as a monodentate bridging group; each PO_4_ group is coordinated to four UO_2_^2+^ ions. A striking structural feature is the arrangement of the water molecules. The size of the hydrogen, alkali and ammonium ionic species in particular compounds affects the content of the crystalline water in the unit-cell.

The unit-cell parameters of alkali uranyl(2+)phosphates are calculated [21,22] from our previously published XRD data [4] and are compared with those of NH_4_[UO_2_PO_4_]·3H_2_O (table 4). Calculated unit-cell dimensions of these compounds are in very good agreement with the values obtained from single-crystal data [30]. The increasing values of the unit-cell volumes of the trihydrates M[UO_2_PO_4_]·3H_2_O, M=Na, K, NH_4_, Rb (table 4), correlate with increasing ionic radii [31] of corresponding species: Na(*r*_i_=0.97 Å), *K*(*r*_i_= 1.33 Å), NH_4_(*r*_i_= 1.43 Å), and Rb(*r*_i_= 1.47 Å).

The results of this work along with the recalculations of previously published experimental results [3,4,14] give a detailed and complete description of the formation, solubility and structural relationship of ammonium and alkali uranyl(2 +)phosphates.

## Figures and Tables

**Table 1:** x-ray powder pattern for NH_4_UO_2_PO_4_·3H_2_O (*P*4*/ncc, a* = 7.02 Å, *c* = 18.08 Å, *Z* = 4)

*h*	*k*	*l*	*d*_obsd_/Å	*d*_calcd_/Å	*I*_rel_
0	0	2	9.08	9.04	100
1	0	2	5.56	5.54	20
1	1	0	4.97	4.96	12
0	0	4	4.53	4.52	12
1	1	2	4.35	4.35	14
1	0	4	3.80	3.80	51
2	0	0	3.51	3.51	17
1	1	4	3.34	3.34	1
2	0	2	3.28	3.27	20
2	1	1	3.09	3.09	1
0	0	6	3.02	3.01	1
2	1	2	2.97	2.97	9
2	1	3	2.78	2.78	29
2	1	4	2.58	2.58	8
2	2	0	2.48	2.48	4
2	2	2	2.39	2.39	5
3	0	2	2.27	2.27	14
3	1	0	2.22	2.22	6
3	1	1	2.21	2.20	3
2	2	4	2.18	2.18	12
3	1	2	2.16	2.16	15
3	1	3	2.08	2.08	6
3	0	4	2.07	2.08	3
1	1	8	2.06	2.06	9
2	1	7	1.998	1.995	1
2	0	8	1.901	1.900	4
3	0	6	1.849	1.848	4
2	1	8	1.834	1.834	5
0	0	10	1.808	1.808	5
3	2	4	1.788	1.788	4
4	0	0	1.754	1.755	1
1	0	10	1.751	1.751	1
4	0	2	1.723	1.723	1
1	1	10	1.699	1.699	12
4	1	1	1.695	1.695	6
2	2	8	1.672	1.671	1
3	3	0	1.655	1.655	1
4	0	4	1.636	1.636	4
3	0	8	1.626	1.626	3
2	0	10	1.608	1.607	6
4	1	4	1.595	1.593	2
3	1	8	1.585	1.584	4
4	2	0	1.570	1.570	1
4	2	2	1.547	1.547	1
0	0	12	1.508	1.507	2
4	2	4	1.484	1.483	1
3	2	8	1.475	1.475	3
2	2	10	1.462	1.461	2
1	1	12	1.442	1.442	6
4	2	5	1.439	1.440	3

**Table 2:** Equilibrium concentrations determined according to precipitation boundary^a^ and calculated *K*_s_ values for NH_4_UO_2_PO_4_·3H_2_O(s)

System no.	10^2^ × *c*(PO_4_)_soln_*/*mol dm^−3^	10^4^ × *c*(NH_4_)_soln_*/*mol dm^−3^	pH	10^2^ × *I*/mol dm^−3^	log *K*_s_	log *K*_s_(*I* = 0)
1	5.0	2.25	1.76	1.90	−26.03	−26.83
2	6.0	4.50	1.71	2.13	−25.84	−26.68
3	8.0	9.00	1.69	2.48	−25.63	−26.52
4	10.0	17.50	1.61	2.89	−25.52	−26.46
5	15.0	40.00	1.53	3.73	−25.40	−26.43
6	20.0	90.00	1.43	4.69	−25.27	−26.39
7	25.0	125.00	1.41	5.40	−25.24	−26.42
8	30.0	175.00	1.33	6.26	−25.26	−26.51
9	40.0	350.00	1.31	8.06	−25.09	−26.44
10	50.0	550.00	1.30	9.95	−24.99	−26.44
11	80.0	1250.00	1.23	15.88	−24.89	−26.54

a In all systems c(UO_2_)_soln_ = 1 × 10^−3^ mol dm^−3^.

**Table 3:** Homogeneous and heterogeneous equilibria^a^

	log *K*(*I* = 0)	Ref.	log *K*(*I* = 0.23 mol dm^−3^)	Ref.
1. H_2_PO_4_^−^ + H^+^ = H_3_PO_4_	2.148 (*K*_13_)	23	2.01	23^e^
2. HPO_4_^−^ + H^+^ = H_2_PO_4_^−^	7.199 (*K*_12_)	24	6.77	24^e^
3. PO_4_^3−^ + H^+^ = HPO_4_^2−^	12.35 (*K*_1_)	25	11.64	25^e^
4. UO_2_^2+^ + H_3_PO_4_ = UO_2_H_2_PO_4_^+^ + H^+^	1.50 (*β*_01_)	5	1.28	5^e^
5. UO_2_^2+^ + H_3_PO_4_ = UO_2_H_3_PO_4_^2+^	1.30 (*β*_10_)	5	1.30	5^e^
6. ${{\text{UO}}_{2}}^{2+}+2H_{3}{\text{PO}}_{4}={\text{UO}}_{2}{\left(H_{2}{\text{PO}}_{4}\right)}_{2}^{0}+2H^{+}$	1.30 (*β*_02_)	5	0.93	5^e^
7. ${{\text{UO}}_{2}}^{2+}+3H_{3}{\text{PO}}_{4}={\text{UO}}_{2}\left(H_{3}{\text{PO}}_{4}\right){\left(H_{2}{\text{PO}}_{4}\right)}_{2}^{0}+2H^{+}$	2.30 (*β*_12_)	5	1.93	5^e^
8. H^+^ + OH^−^ = H_2_O	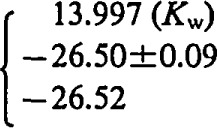	25	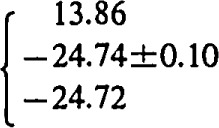	25^e^
9. NH_4_UO_2_PO_4_·3H_2_O(s) = NH_4_ + UO_2_^2+^ PO_4_^3−^		b		c
		c,f		b,e
10. NaUO_2_PO_4_·3H_2_O(s) = Na^+^ + UO_2_^2+^ +PO_4_^3−^	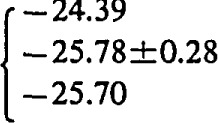	c,f	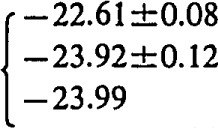	c
11. KUO_2_ PO_4_·3H_2_O(s) = K^+^ + UO_2_^2+^ +PO_4_^3−^		d		c
		c,f		d,e
12. RbUO_2_PO_4_·3H_2_O(s) = Rb^+^ + UO_2_^2+^ + PO_4_^3−^	−25.91	c,f	−24.13±0.19	c
13. CsUO_2_PO_4_·2·5H_2_O(s) = Cs^+^ + UO_2_^2+^ +PO_4_^3−^	−25.59	c,f	−23.80±0.20	c

a At 298 K.

b This work.

c Recalculated ill this work from reference 14.

d Recalculated in this work from reference 3.

e Corrected from *I* = 0 to *I* = 0.23 mol dm^−3^.

f Corrected from, *I* = 0.23 mol dm^−3^ to *I* = 0.

**Table 4:** Unit-cell parameters (space group *P*_4_/*ncc, Z* = 4) of hydrogen, ammonium and alkali uranyl(2+) phosphates

Compound	*a*/Å	*c*/Å	*V*/Å^3^
H_3_O[UO_2_PO_4_]·3H_2_O [15]	6.995	17.491	855.84
Li[UO_2_PO_4_]·4H_2_O	7.04(2)	18.28(7)	906.0
Na[UO_2_PO_4_]·3H_2_O	7.01(2)	17.52(4)	860.9
K[UO_2_PO_4_]·3H_2_O	7.01(1)	17.84(6)	876.7
NH_4_[UO_2_PO_4_]·3H_2_O	7.02(1)	18.08(4)	891.0
Rb[UO_2_PO_4_]·3H_2_O	7.00(1)	18.36(5)	899.6
Cs[UO_2_PO_4_]·2·5H_2_O^a^	7.06(2)	17.80(8)	887.2

a Pseudo tetragonal (monoclinic) [4,30].

## Footnotes

2 Certain commercial materials and equipment are identified in this paper to specify the experimental procedure. In no instance does such identification imply recommendation or endorsement by the National Bureau of Standards or the ADA Health Foundation or that the material or equipment identified is necessarily the best available for the purpose.
